# Dynamic Multibody Modeling of Spherical Roller Bearings with Localized Defects for Large-Scale Rotating Machinery

**DOI:** 10.3390/s25082419

**Published:** 2025-04-11

**Authors:** Luca Giraudo, Luigi Gianpio Di Maggio, Lorenzo Giorio, Cristiana Delprete

**Affiliations:** Dipartimento di Ingegneria Meccanica e Aerospaziale (DIMEAS), Politecnico di Torino, Corso Duca Degli Abruzzi 24, 10129 Torino, Italylorenzo.giorio@polito.it (L.G.)

**Keywords:** condition monitoring, rolling element bearings, multibody model, predictive maintenance, fault detection, machine diagnosis, rotating machinery, fault detection, mechanical vibration

## Abstract

Early fault detection in rotating machinery is crucial for optimizing maintenance and minimizing downtime costs, especially in medium-to-large-scale industrial applications. This study presents a multibody model developed in the Simulink^®^ Simscape environment to simulate the dynamic behavior of medium-sized spherical bearings. The model includes descriptions of the six degrees of freedoms of each subcomponent, and was validated by comparison with experimental measurements acquired on a test rig capable of applying heavy radial loads. The results show a good fit between experimental and simulated signals in terms of identifying characteristic fault frequencies, which highlights the model’s ability to reproduce vibrations induced by localized defects on the inner and outer races. Amplitude differences can be attributed to simplifications such as neglected housing compliancies and lubrication effects, and do not alter the model’s effectiveness in detecting fault signatures. In conclusion, the developed model represents a promising tool for generating useful datasets for training diagnostic and prognostic algorithms, thereby contributing to the improvement of predictive maintenance strategies in industrial settings. Despite some amplitude discrepancies, the model proves useful for generating fault data and supporting condition monitoring strategies for industrial machinery.

## 1. Introduction

Early detection of malfunctions in rotor systems can lead to significant economic benefits in the maintenance strategies of industrial systems operating in manufacturing settings [1,2]. In particular, it is well known that sensing and interpretation of signals from rotating machinery fosters condition-based maintenance [3] through analysis of the vibratory, thermal, and lubrication parameters of rolling bearings, which emerge as key elements of rotating machinery [4,5].

Over the past decade, development of data-driven diagnosis and maintenance methodologies has attracted increasing interest in the scientific literature due to the high performance achievable through machine learning and deep learning techniques [6,7,8,9], allowing for the identification of patterns and classification of data to exploit learning on appropriate training datasets. However, in the field of rotating system diagnosis it is particularly challenging to obtain a comprehensive dataset representing the different operating conditions of the machine, including its various damage modes. To address this critical issue, a growing body of research is focusing on strategies to fill the gaps in available data [10], including knowledge transfer [11,12,13,14,15] domain adaptation approaches [16,17], data augmentation techniques [18,19], and Generative Adversarial Networks (GANs) [20,21,22,23]. In addition, the use of simulation models to generate databases represents a promising approach, potentially complementing or even replacing entire experimental campaigns. Several simulation methodologies [24,25,26] have been adopted to approach mathematical formulations capable of representing the nonstationary behavior of bearing signals [27].

In this context, Vehviläinen et al. [28] developed a multibody simulation system incorporating a geometry-based polygonal contact method to accurately capture nonlinear dynamics and simulate faulty bearings with local outer ring faults [29]. They leveraged 3D multibody simulations to generate a comprehensive dataset of healthy and faulty bearings with realistic defects, facilitating data-driven fault diagnosis and machine learning-based classification with high accuracy. Gismeros Moreno et al. [30] proposed a smooth-contact formulation for modeling radial-loaded deep groove ball bearings under multibody dynamics, focusing on cage/rolling element interaction. Tian et al. [31] built a 4-DOF dynamic model of an inter-shaft bearing with local defects considering Elasto-Hydrodynamic Lubrication (EHL), while Luo et al. [32] proposed a 4-DOF dynamic model of a Rolling Element Bearing (REB) with compound faults of both the inner and outer raceway surface. They considered the time-varying displacement, impact force excitation, and EHL conditions, introducing an excitation function with improved impact forces. Liu and Shao [33] presented a comprehensive review of dynamic modeling and analysis methods for predicting the vibration characteristics of REBs with and without localized and distributed faults. Guo et al. [34] proposed a dynamic model of a rolling bearing variable stiffness system with local faults to consider the retention factor of the contact deformation. Zhang et al. [35] considered cage flexibility, local faults, and EHL to reflect the force states and vibration responses of rolling bearings. Nakhaeinejad and Bryant [36] simulated the multibody dynamics of healthy and faulty REBs using vector bond graphs, which they modeled using a 33-DOF model. Li et al. [37] proposed a dynamic simulation-driven fault diagnosis framework using a Security Transfer Support Matrix Machine (STSMM) model to address transfer learning issues in rolling bearings and avoid negative transfer. Xiong et al. [38] developed a 5-DOF composite fault model of rolling bearings considering EHL, whereas Zhang et al. [39] proposed a numerical model-driven cross-domain fault diagnosis method based on the Cross-Domain Nuisance Attribute Projection (cDNAP). Savas et al. [40] simulated multiple defects in ball bearings for aerospace applications, while Zhao et al. [41] proposed a basic modeling framework for bearing fault simulations. In this approach, a three-dimensional mode is established according to the geometric size of the physical entity, then a virtual fault is injected into the virtual simulation model to generate fault characteristics through model simulation.

Despite numerous studies in the literature, most of the proposed models concern small rolling bearings, for which experimental data from public datasets are often available [4,42,43]. On the other hand, there are no specific models for medium to large spherical roller bearings, which are of particular interest for industrial applications [44,45,46,47], especially in the area of heavy rotating industrial machinery. In addition, damage modeling with variable geometries to simulate different degradation scenarios in these bearings remains an under-explored aspect. In particular, the effect of rolling elements and their inertial properties is often neglected, and slippage of the rolling elements is not considered. As a result, the generation of periodic defect-induced pulses is not always representative of actual bearing behavior.

To address these shortcomings, this paper proposes the development of a multibody model for medium to large spherical roller bearings. The model is implemented in Simulink^®^ Simscape Multibody (version 2023, Mathworks®) and considers all DOFs of the bearing’s solid elements, allowing for a more realistic description of the dynamic behavior. Simulation results are compared with experimental data obtained from a test campaign performed on a test rig for medium to large industrial bearings located at the Mechanical Engineering Laboratories of the Politecnico di Torino [48,49]. To the best of the authors’ knowledge, this is the first study in the literature to present a multibody model for medium to large spherical roller bearings and to experimentally validate it through data acquired from real tests.

Having reviewed the literature on dynamic simulation of bearing systems and the innovative contributions of this work in the above introductory section, the remainder of this paper is organized as follows: Section 2 presents the proposed multibody model and its formulation along with the contact model, simulation parameters, and simulation framework; Section 3 introduces the experimental activity and the dataset related to spherical roller bearings with localized damage; Section 4 presents the results and analysis; Section 5 provides a discussion of the findings; finally, Section 6 presents the conclusions.

## 2. Multibody Dynamic Model for Spherical Roller Bearings with Faults

**Figure 1:**
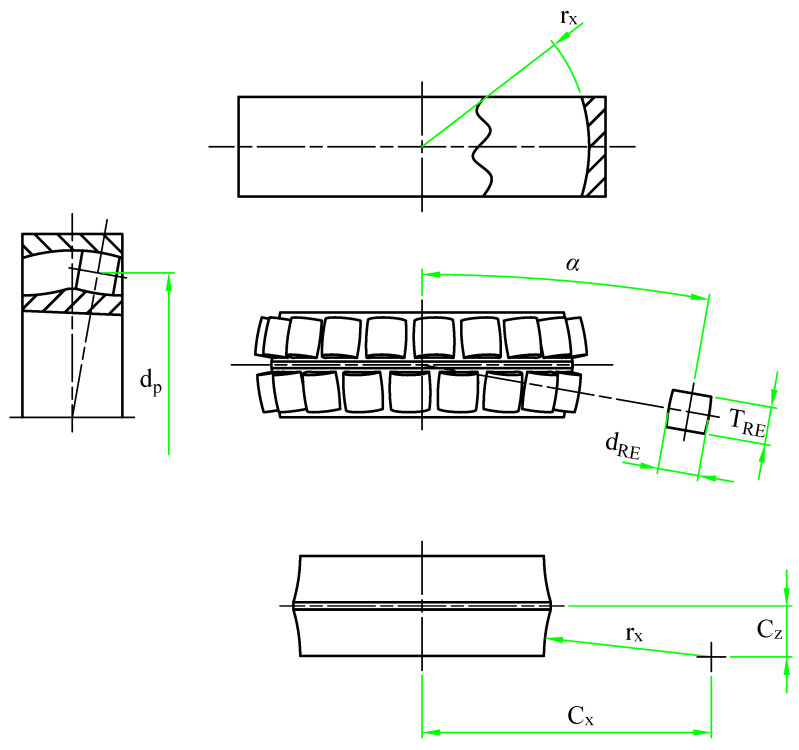
Fundamental geometry of the bearing model.

In order to investigate the dynamic behavior of spherical roller bearings and evaluate the impact of localized defects, a multibody simulation model was developed in Simulink^®^ Simscape Multibody. This model depicts the relationships among the Inner Race (IR), Outer Race (OR), Rolling Elements (RE), and cage, with the integration of essential geometric features shown in Figure 1 and outlined in Table 1.

In order to provide a more accurate dynamic response, the model was developed to incorporate all DOFs of the bearing parts. The inertial characteristics were obtained from CAD models. The numerical implementation was performed on a high-performance computing system, with an explicit solver utilized to simulate large displacements. The model was adjusted and validated using the experimental campaign presented in Section 3. Considering the aim of the simulation analyses included in this paper, the nonlinearities in the overall system were considered not to introduce chaotic effects. From the perspective of machine diagnosis, sensitivity to changes in initial conditions does not affect the detectability of defects; therefore, for the purpose of this study, we consider this phenomenon negligible.

This section outlines the multibody modeling strategy, simulation structure, and numerical methods used to emulate the physical behavior of spherical roller bearings with localized defects.

### 2.1. Multibody Model Formulation

The model was implemented in Simulink^®^ using the Simscape Multibody library (version 2023, Mathworks®). As this software does not explicitly disclose the underlying multibody formulation, a Lagrangian-based approach (Equation (1)) is assumed. This formulation is commonly used for flexible and constrained systems. Here, [M] represents the mass matrix, [Φ] is the Jacobian of the constraints, {λ} denotes Lagrangian multipliers, *Q* denotes the generalized forces acting on the system, and $\left\{\ddot{q}\right\}$ represents the accelerations of the generalized coordinates.

(1) $$\left[\begin{array}{cc} \left[M\right] & {\left[\Phi \right]}^{T}\\ \left[\Phi \right] & \left[0\right] \end{array}\right]\left\{\begin{array}{c} \left\{\ddot{q}\right\}\\ \left\{\lambda \right\} \end{array}\right\}=\left\{Q\right\}$$

The Reference Frames (RFs) establish the positioning and movement limitations between bodies via joints. Each body inherently has an RF, which is defined by either its geometric construction or its initial position within the simulation. Each initial RF is defined by Cartesian coordinates and an angular orientation relative to the geometry of the body. In this framework, the DOFs for every initial RF are specified as follows:

(2) $$\left\{x\right\}=\left\{\begin{array}{c} x\\ y\\ z\\ \varphi_{x}\\ \varphi_{y}\\ \vartheta \end{array}\right\}$$

where x,y,z are translational DOFs and $\varphi_{x},\varphi_{y},\vartheta$ are rotational DOFs associated with the axis in the same order. In this work, the subscripts indicate the corresponding body.

New RFs can be established by utilizing coordinate transformations that alter their position and orientation. A World Frame (WF) is created as the universal inertial reference, acting as the Newtonian coordinate system for the model. Forces and torques can only be applied on RFs, indicating that external forces impacting a particular point of a body require either the establishment of a new reference system at the point of force application or breaking down of the applied force into normal and tangential components concerning a given RF.

The bearing model contains 41 total bodies, including 38 REs along with the inner ring, outer ring, and cage. Each element has its original RF in the center of revolution, with the *z* axis used as the revolution axis. The xy axis defines the plane of symmetry corresponding to the raceways of both the IR and OR, while in the REs it is the symmetry plane parallel to the non-contacting faces.

In the multibody model, each part of the bearing is assigned particular kinematic constraints and DOFs in order to faithfully reproduce its dynamic behavior. The RFs and the transformations that establish these constraints are detailed below.

**Figure 2:**
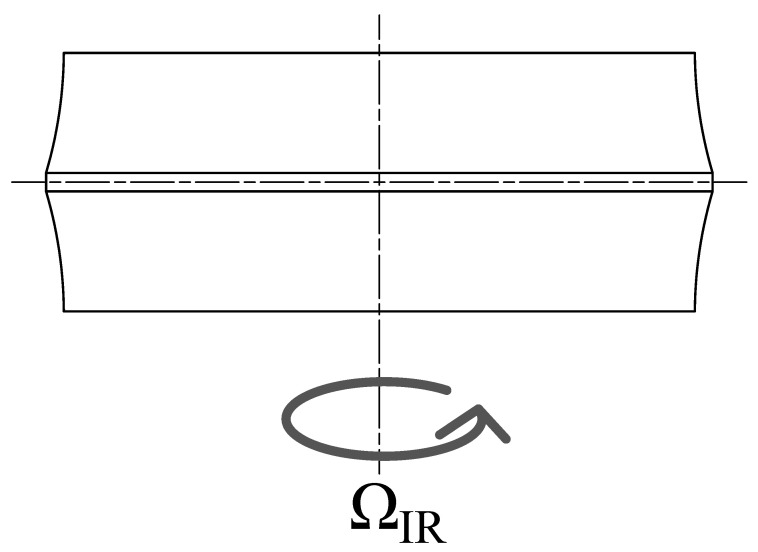
Schematic representation of the DOFs of the inner ring.

**Figure 3:**
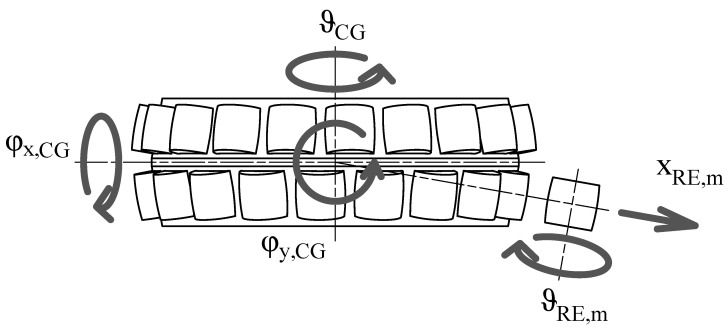
Schematic representation of the DOFs of the cage and REs in the initial condition.

**Figure 4:**
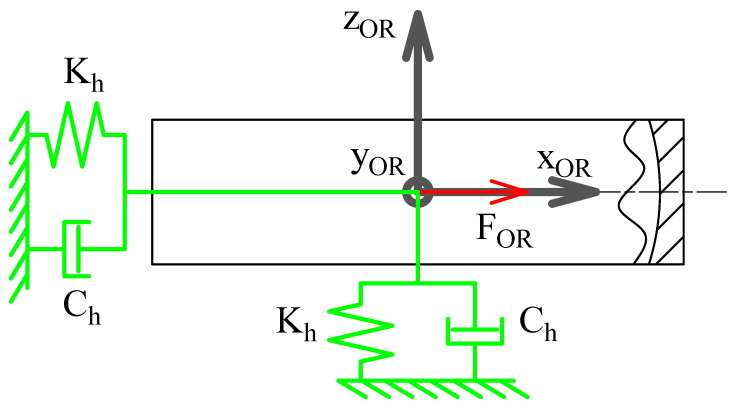
Schematic representation of the DOFs of the outer race.

- The inner race is coupled to the WF by five DOFs, leaving only the rotation ϑ around the *z* axis free. The desired rotational speed $\Omega_{IR}=\frac{d\vartheta }{dt}$ is then specified through a data block, which can be set as either a constant or a function variable in time (Figure 2). In order to represent the test rig configuration, the shaft stiffness is hypothesized to be extremely high compared than the system under simulation.
- Each RE is positioned in a specific slot in the cage by rotating on the *z* axis, tilting on the *y* axis, and translating the cage RF by $\vartheta_{CG,m}=\frac{2\pi }{M}$, α, and $\frac{d_{p}}{cos(\alpha )}$, respectively, where *m* is used to represent the *m*th RE, as *M* is the number of REs per row. The operations are executed with an axis rotation, a quaternion rotation, and a cylindrical transform.
  The rotation’s formulation is as follows:
  (3) $${\left\{x\right\}}_{CG,m}=\left[\begin{array}{cc} \left[A\right] & \left[0\right]\\ \left[0\right] & \left[A\right] \end{array}\right]\left\{x\right\}$$
  where {x} are the base DOFs. As in Equation (2), [0] is a 3×3 zeroes matrix and [A] is the following rotation matrix:
  (4) $$\left[A\right]=\left[\begin{array}{ccc} cos(\vartheta_{CG,m}) & sin(\vartheta_{CG,m}) & 0\\ -sin(\vartheta_{CG,m}) & cos(\vartheta_{CG,m}) & 0\\ 0 & 0 & 1 \end{array}\right]$$
  where the subscript CG,m indicates the *m*th slot in the cage.
  Tilting by α is applied as a quaternion rotation. The elements in the ${\left\{x\right\}}_{CG,m}$ vector are translated into the complex space to create quaternions with the following arrangement:
  (5) $$\begin{array}{cc} 0+ix_{CG,m} & +jy_{CG,m}+kz_{CG,m} \end{array}$$
  (6) $$\begin{array}{cc} 0+i\varphi_{x,CG,m} & +j\varphi_{y,CG,m}+k\vartheta_{CG,m} \end{array}$$
  where i,j,k are basis unit vectors in the complex space. The two DOFs are then subjected to the following quaternion rotation:
  (7) $$\begin{array}{cc} X_{CG,m} & =\bar{q}\;\left(0+ix_{CG,m}+jy_{CG,m}+kz_{CG,m}\right)\;q \end{array}$$
  (8) $$\begin{array}{cc} \Theta_{CG,m} & =\bar{q}\;\left(0+i\varphi_{x,CG,m}+j\varphi_{y,CG,m}+k\vartheta_{CG,m}\right)\;q \end{array}$$
  where *q* is the quaternion and $\bar{q}$ is its complex conjugate, defined as follows:
  (9) $$q=cos\left(\frac{\alpha }{2}\right)+i\hat{x}sin\left(\frac{\alpha }{2}\right)+j\hat{y}sin\left(\frac{\alpha }{2}\right)+k\hat{z}sin\left(\frac{\alpha }{2}\right)$$
  where $\hat{x},\hat{y},\hat{z}$ are the components of the unit vector indicating the direction parallel to the tilt rotation, which in our case is
  (10) $$\left\{\begin{array}{c} \hat{x}\\ \hat{y}\\ \hat{z} \end{array}\right\}=\left\{\begin{array}{c} 0\\ 1\\ 0 \end{array}\right\}.$$
  Finally, the vector is recomposed and translated to the correct radial position:
  (11) $${\left\{X\right\}}_{CG,m}^{\prime }={\left\{X\right\}}_{CG,m}+\left\{t\right\}$$
  where {t} is
  (12) $$\left\{t\right\}=\left\{\begin{array}{c} \frac{d_{p}}{cos(\alpha )}\\ 0\\ 0\\ 0\\ 0\\ 0 \end{array}\right\}.$$
  This leaves us with an RF defined as
  (13) $${\left\{X\right\}}_{CG,m}^{\prime }=\left\{\begin{array}{c} X_{CG,m}^{\prime }\\ Y_{CG,m}^{\prime }\\ Z_{CG,m}^{\prime }\\ \Phi_{x}^{\prime }\\ \Phi_{y}^{\prime }\\ \Theta_{CG,m}^{\prime } \end{array}\right\},$$
  which we couple with a pin slot joint to the *m*th RE’s degrees of freedom:
  (14) $${\left\{x\right\}}_{RE,m}=\left\{\begin{array}{c} x_{RE,m}\\ y_{RE,m}\\ z_{RE,m}\\ \varphi_{x}\\ \varphi_{y}\\ \vartheta_{RE,m} \end{array}\right\}$$
  which leaves the translation perpendicular to the cage slot and the rotation $\varphi_{RE,m}$ both free, as is reproduced in Figure 3. The perpendicular translation, which at time 0 is $x_{RE,m}$, changes during simulation due to rolling of the RE, meaning that the actual coupling condition is as shown below.
  (15) $$\left\{\begin{array}{c} Y_{CG,m}^{\prime }-\left(x_{RE,m}\;sin\left(\vartheta_{RE,m}-\Theta_{CG,m}\right)+y_{RE,m}\;cos\left(\vartheta_{RE,m}-\Theta_{CG,m}\right)\right)=0\\ Z_{CG,m}^{\prime }-z_{RE,m}=0\\ \Phi_{x,CG,m}^{\prime }-\varphi_{x,RE,m}=0\\ \Phi_{x,CG,m}^{\prime }-\varphi_{y,RE,m}=0 \end{array}\right.$$
  This coupling condition simulate the possible movements of the REs in the cage without modeling contacts, which simplifies the calculations and reduces the computation time. It is hypothesized here that the effects of the cage with regard to RE contact can be neglected and that the vibrational contributions of said contact are not significant for diagnosis of the type of faults under investigation. In addition, it is assumed that the RE cannot tilt in the cage’s slot, which binds together the DOFs from the RFs of the two bodies.
- The cage is coupled to the inner ring RF trough a gimbal joint, which leaves the three rotations free. This model neglects the contact between the CG and IR, which although relevant for IR-guided cages is not significant enough to justify explicit modeling.
- The outer race is joined to the WF by locking the three rotations while leaving the translations free. The effect of the housing is modeled as a spring damper with stiffness $K_{h}$ and damping $C_{h}$. To represent the housing inertia, a point mass $m_{h}$ is added to the OR. An external force $F_{OR}$ is also applied to the RF of the outer race. This is schematically represented in Figure 4.

### 2.2. Contact Model

Simscape Multibody provides multiple contact models, summarized in Table 2. To represent REs, the model uses Convex Hull (CH) from a CAD file. The disk model creates a 2D circular disk that can be used for contact modeling. For the aims of this study, this model was excluded in order to keep the representations of the contact surfaces of the IR and the OR equivalent in terms of geometrical representation; indeed, multiple disks cannot be used to represent the outer race. The grid surface represents a topological map provided in the form of xyz point coordinates. The software then creates surfaces connecting these points. Because two different *z* coordinates cannot exist for the same xy point, it would be necessary to create separate grids for each raceway.

Although it would have been feasible to utilize this formulation in conjunction with the point cloud block to simulate REs, we initially chose to forgo this approach in order to avoid the need for multiple grid partitions. Given that the raceways possess an inherently concave geometry, the application of CAD geometry was rendered impractical, as the library exclusively accommodates contact calculations subsequent to the implementation of a CH approximation. As a result, the only viable methodology within the Simscape Multibody framework was to depict the raceways through the point cloud technique while utilizing CH geometry for the REs.

Each raceway is represented by three circumferences made of $n_{pc}$ discreet points, creating a angular resolution of

(16) $$\Delta \vartheta =\frac{2\pi }{n_{pc}},$$

with Δϑ being the angular distance between each point. This quantity describes how the system acts when rotating. A high-frequency excitation is expected due to the REs falling into the gaps between the discretized points, similar to the excitation observed in mesh-based models when two meshed objects roll over each other [50]. The circumferential points are defined based on the raceway geometry extracted from CAD models, with their Cartesian coordinates computed as follows:

(17) $$\left\{\begin{array}{c} x_{k}\\ y_{k}\\ z_{k} \end{array}\right\}=\left\{\begin{array}{cc} \left\{\begin{array}{c} (C_{x}+r_{x}cos\left(p\alpha \right))sin\left(k\Delta \vartheta \right)\\ (C_{x}+r_{x}cos\left(p\alpha \right))cos\left(k\Delta \vartheta \right)\\ \left(C_{z}-r_{x}sin\left(p\alpha \right)\right) \end{array}\right\}\; & \;\mathrm{for}\;\mathrm{the}\;\mathrm{IR}\;\mathrm{pointcloud}\\ \left\{\begin{array}{c} (r_{x}cos\left(p\alpha \right))sin\left(k\Delta \vartheta \right)\\ (r_{x}cos\left(p\alpha \right))cos\left(k\Delta \vartheta \right)\\ \left(r_{x}sin\left(p\alpha \right)\right) \end{array}\right\}\; & \;\mathrm{for}\;\mathrm{the}\;\mathrm{OR}\;\mathrm{pointcloud} \end{array}\right.$$

where k indicates the *k*th point and *p* is a vector

p=[0.5,1,1.5]

that distinguishes each of the three circumferences. Here, α is equal to ±10, with the duality of the sign used to represent the two symmetrical raceways.

The RE is acquired from a .step ap214 file. Enhanced control over the contact simulation can be realized through the use of an .stl file, which allows alterations to the triangle density. Specifically, diminishing this parameter can enhance computational efficiency and result in reduced simulation duration, albeit with a concomitant reduction in model accuracy. Considering that the precise geometric characteristics of the bearing remain indeterminate, the model was constructed without factoring in the curvature variations typically observed between raceways and REs in this category of bearing.

**Figure 5:**
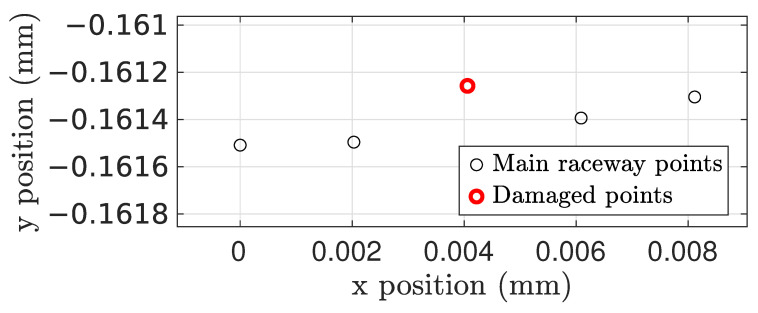
Example of a single point damage representation on the central circumference of the outer ring’s raceway.

Localized faults were modeled independently on the inner and on the outer races. Damage can be activated when desired, and is represented by moving a series of points in the central circumference of the point cloud. We modeled damage in the positive normal direction to the surface, as this ensures that the fault is actually significant [51]. The fault can be represented by either modifying the radial position of a number $n_{d}$ of existing points or by adding new ones; as an example, Figure 5 shows a single point damage example with a 0.2 mm positive offset fault on the outer ring.

Because one of the geometries used in the model is represented as a set of discrete points rather than by using continuous surfaces, directly computing the contact pressure is not feasible. While we acknowledge the existence of methodologies for estimating pressure from nodal forces, these approaches would provide only an approximate solution if applied to the results from this model. Therefore, we decided not to include them in the current study. As a direct consequence of this, pressure-based contact force formulations such as EHL models cannot be implemented at this stage of model development. Instead, the current approach determines the contact force based on the penetration depth between the two geometries. The penetration depth is calculated for each point of the point cloud. The depth is positive when a point is inside the CH of an RE. The contact forces are calculated normal to the penetration depth δ and penetration velocity $\dot{\delta }$, as reported in Equation (18):

(18) $$F_{c,n}=\left\{\begin{array}{cc} K_{c}\delta +C_{c}\dot{\delta } & \mathrm{if}\;\delta \geq w\\ s\left(\delta ,w\right)\left(K_{c}\delta +C_{c}\dot{\delta }\right) & \mathrm{if}\;0\leq \delta <w\\ 0 & \mathrm{if}\;\delta <0 \end{array}\right.$$

where s(δ,w) is a smoothing function used by the software to reduce impulsive-like behavior due to contacts between fast-moving objects. This function acts when δ is between zero and *w* and increases monotonically in this range from 0 to 1. Its derivative is equal to zero at the endpoints and the *w* parameter is set to $10^{-8}$, which is three orders of magnitude below the standard penetration depth.

The frictional force model operates tangentially to the previous formulation, generating a force opposite to the direction of relative velocity at the contact point. The modulus is provided by

(19) $$|F_{c,t}\left|=\mu \right|F_{c,n}|,$$

where μ is the coefficient of friction. This value changes depending on the relative tangential velocity between the two contacting bodies. The relation is formulated as a classic Coulomb one in which the effective coefficient of friction varies between two values, with $\mu_{s}$ being static and $\mu_{d}$ being dynamic.

The model used to represent the contact force is of the Kelvin–Voigt type, which describes viscoelastic behavior. Although alternative models may provide a more accurate representation of energy losses, a simple model appropriate for solid materials was chosen at this preliminary stage. The Kelvin–Voigt model is relatively easy to implement and provides a reasonable description of viscoelastic behavior for initial applications. At this stage, a sufficiently descriptive approach was preferred, without introducing excessive complexity that would have resulted in an increase in the computational resources required; adopting this model simplifies the analysis while maintaining an adequate level of accuracy for the simulations envisioned at this early stage.

The limits of such a modeling approach are bounded by the use of a linear contact model within the hypothesis of linear elastic range. As the model was developed, it was also noted that the model starts to produce an incorrect output after a certain rotational speed. The reason for this is possibly due to using too low of a timestep. If the timestep is not sufficiently small, this could render the contact calculation unstable. If the speed of the IR grows too high, the penetration δ varies erratically, as the position of the IR point cloud at each timestep changes too quickly to allow for correct representation of contact events. For this reason, the model was not simulated for speeds above 1000 RPM.

### 2.3. Simulation Parameters and Computational Setup

**Figure 6:**
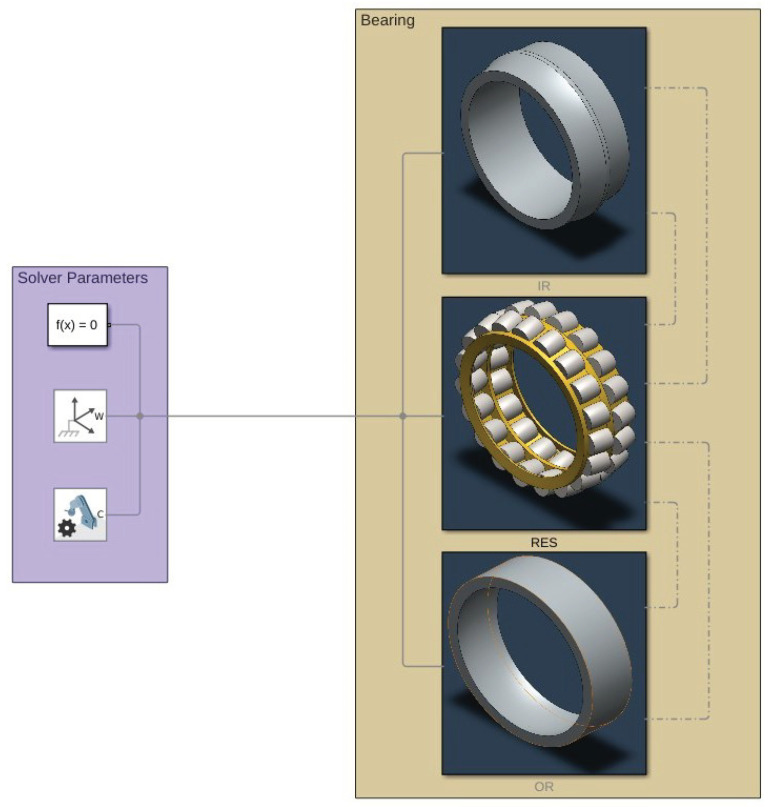
Overlook of the Simulink^®^ model.

**Figure 7:**
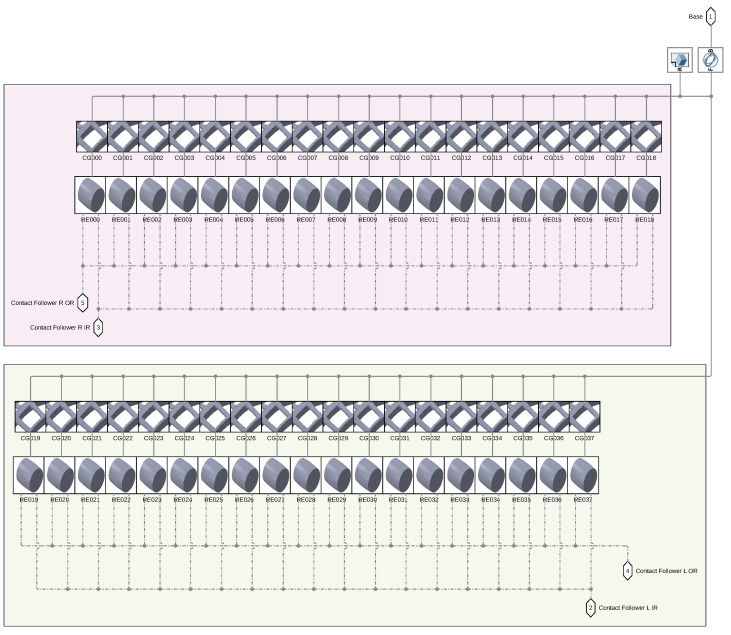
Inside view of the RES subsystem.

**Figure 8:**
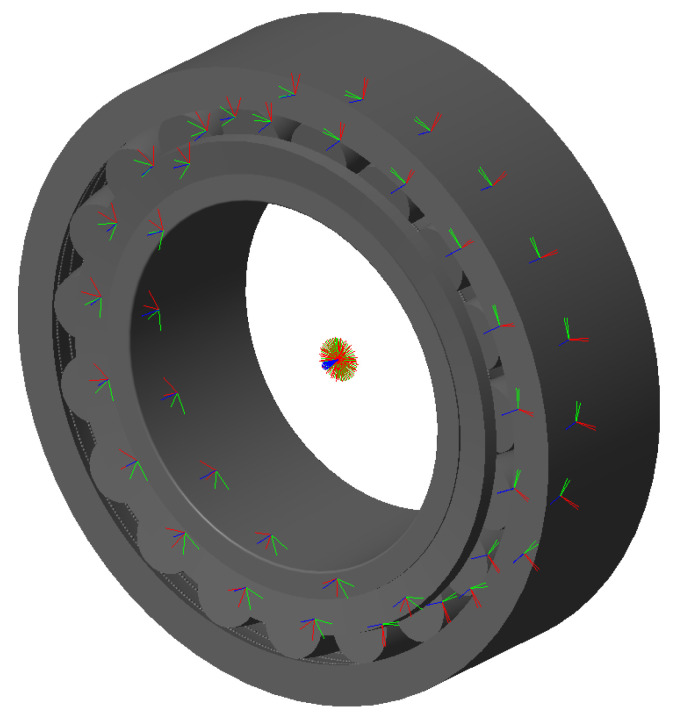
Bearing model graphics in the initial phase of rolling.

The Simulink^®^ model is represented in Figure 6, Figure 7 and Figure 8. The model is mainly divided in three larger subsystems that contain the multibody modeling blocks. Contact data are passed between rings and rolling elements, where force calculations are computed.

As can be seen in Figure 7, each rolling element is connected to the relative cage slot and to the two raceway geometries.

Simulations were run using the ODE4 solver with a $1.628\times 10^{-5}$ s fixed timestep. ODE4 was found to be the lowest order of solver that could stably compute the model. Indeed, the use of an explicit solver creates another stability condition, which forces the use of a timestep lower than a certain value, above which the time integration becomes unstable and the solution diverges.

The Simulink^®^ model was coded using the Simulink^®^ coder into .c code files, which were then compiled to run in an executable. Different rotational speeds could then be simulated in parallel using the High-Performance Computing (HPC) service provided by Politecnico di Torino (Torino, Italy) (HPC@POLITO). The characteristics of the hardware are reported in Table 3.

Computational times were on the order of 1.26 s/day and varied depending on the external load, rotational speed, and geometrical resolution. The model was simulated to obtain signals of approximately ten seconds. We note that the code used to calculate the contact forces was relatively unoptimized; indeed, the .c files reveal that the code constructs contact data sequentially before passing it to the function responsible for the force computation. As a result, the contact force for each of the 38 REs (REs) is calculated one at a time in a serial manner. In the authors’ opinion, this approach may introduce inefficiencies, as the contact forces are independent at each timestep and as such could be computed in parallel to improve computational performance. The parameters of the simulation can be found in Table 4.

## 3. Experimental Framework and Dataset

To validate the proposed model and assess its ability to simulate actual operating conditions, we exploited an experimental test campaign conducted on a test rig specifically designed for medium-sized bearings. This experimental configuration facilitates the systematic collection of vibration data across different load scenarios, offering standard data for assessing the dynamic performance of damaged bearings. The test setup enables the application of both radial and axial forces while rotating at various spin speeds. The collected dataset comprises vibration signals associated with various bearing health conditions, enabling a thorough evaluation of localized defects. This section outlines the experimental test setup utilized for data collection and offers a summary of the benchmark dataset used in this research.

### 3.1. Test Rig for Medium-Sized Bearings

**Figure 9:**
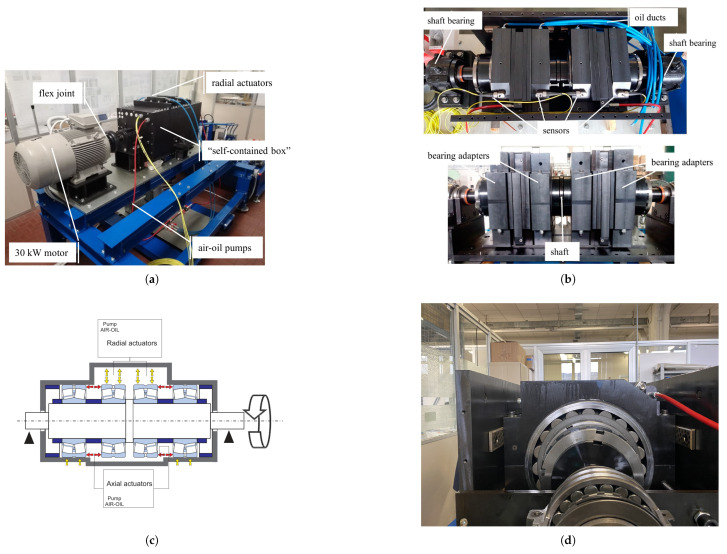
Test rig used for condition monitoring of medium-sized bearings: (**a**) self-contained box [48]; (**b**) shaft, adapters, and sensors [48]; (**c**) load path [48]; (**d**) bearing SKF^®^ 22240 CCK/W33.

The test rig employed in the present investigation is situated at the Politecnico di Torino and has been engineered to assess medium to large industrial bearings under controlled conditions. The apparatus showed in Figure 9a is designed for the systematic acquisition of vibrational data. The key characteristics of the test rig are outlined in this section, with further details available in [48].

The configuration accommodates bearings with outer diameters ranging from 280 mm to 420 mm, allowing for the application of variable radial and axial loads. The modular architecture shown in Figure 9b of this setup allows for interchangeability of bearing dimensions, rendering it adaptable to a wide array of operational contexts. A 30 kW SIEMENS^®^ electric motor modulated through an inverter drives the primary shaft by a PERIFLEX^® elastic coupling^, ensuring transmission of torque. The test rig is characterized by a self-contained structure in which externally imposed loads are counterbalanced through the elastic deformation of the box, obviating the necessity for oversized support bearings. The load path is shown in Figure 9c. Hydraulic actuators are employed for application of independent radial and axial forces of up to 200 kN, with power provided by air–oil conversion pumps integrated into the laboratory’s pneumatic infrastructure. Bearings are secured within custom-engineered adapters that guarantee direct force transmission and regulated lubrication. A recirculating lubrication system delivers ISO VG 150 of oil at a rate of 2.5 L/min under a pressure of 6 bar. Vibration monitoring is executed via SKF^® CMSS 2200T piezoelectric accelerometers^, which are affixed to the bearing adapters and interfaced with an LMS^® Scadas data acquisition system^. The supervision of real-time testing is conducted through TestLab software. In the context of this study, the SKF^® 22240 CCK/W33 spherical roller bearing^ shown in Figure 9d was subjected to testing.

The experimental apparatus described above introduces several pivotal innovations. The platform is specifically dedicated to assessing medium to large bearings subjected to systematically controlled loading conditions. Independent load control mechanisms are provided, emulating real-world scenarios encountered in heavy industries such as rolling mills and paper manufacturing. A modular design is exploited to facilitate flexible experimentation across a range of bearing sizes and industrial applications.

### 3.2. Benchmark Dataset for Localized Faults in Spherical Roller Bearings

This study uses a subset of the vibration dataset acquired through the test rig [49] as a representative systematic compilation of vibrational data related to medium to large industrial bearings. In contrast to established datasets that predominantly focus on small bearings under constrained operational circumstances, this dataset holds particular significance for industrial applications that are not considered in the fault data usually reported in the literature.

The dataset employed as a reference evaluates three distinctive health states for the SKF^®^ 22240 CCK/W33 spherical roller bearing. This bearing is characterized by an inner diameter of 200 mm, a 1:12 tapered bore, and an outer diameter of 360 mm. The experimental conditions encompass the following:

- H (Healthy), representing the nominal condition devoid of any damage.
- IR (Inner Race), representing the presence of damage to the inner raceway.
- OR (Outer Race), representing the presence of damage to the outer raceway.
- B (Rolling Element Defect), representing the presence of damage to one of the rolling elements.

**Figure 10:**
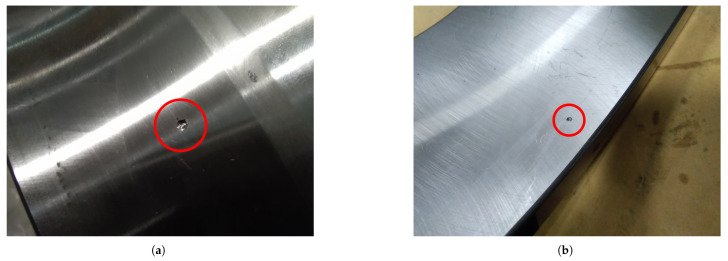
Localized damage: (**a**) IR damage and (**b**) OR damage [14].

Faults were introduced through mechanical machining. Specifically, a solid carbide drill with a diameter of 2 mm was used to produce localized defects measuring 2 mm in diameter and 0.5 mm in depth. The examples of damage shown in Figure 10 were produced on the most heavily loaded raceway under axial loading conditions. Although these defects serve as representations of localized faults in rolling bearings, it is imperative to acknowledge that the derived vibrational data do not encompass the entire array of potential bearing defects.

The dataset includes four distinct load cases, wherein both radial and axial loads were independently regulated through hydraulic actuators as shown in Table 5. The dataset spanned a comprehensive spectrum of rotational speeds, mirroring various industrial operational scenarios. Vibrational signals were acquired for a duration of 30 s per test employing a sampling frequency of 20,480 Hz.

The analyses conducted in this study each focus on a specific load condition, allowing the proposed methodology to be validated using a restricted subset of the dataset while excluding the effects of axial loads. Specifically, we maintain a fixed radial load of 124.8 kN, facilitating our examination of fault patterns at varying speeds while mitigating confounding effects arising from the interplay between load and speed variability. To investigate vibrational signals within the time–frequency domain, the Continuous Wavelet Transform (CWT) was employed [2,52].

## 4. Results

This section presents and analyzes the outcomes of the simulated and experimental signals collected from REBs experiencing various fault conditions. The aim is to assess the capabilities of the multibody model in capturing the primary vibration patterns linked to damage in the Outer Ring (OR) and Inner Ring (IR) as well as to confirm its reliability by contrasting the findings with experimental data obtained from the test rig. The examination is performed for the OR, IR, and H conditions. For each condition, we examine the acceleration signals and their envelopes (the Envelope Spectrum and CWT spectrum). For the sake of conciseness, a subset of the results corresponding to the specific nominal rotational speed of 710 rpm is reported and discussed in detail. In conducting the frequency analysis, we rely on the existing literature, where the use of accelerometer signals for fault detection is commonly accepted as standard [1,2,4], which is also in accordance with what can be measured experimentally. In this particular case, the use of envelope-type spectra allows the characteristic frequencies of the defect to be highlighted by identifying the dominant frequencies in the demodulated signal, which are obtained by processing the signal itself.

The model provides the simulation along with the three axial acceleration components of the Outer Ring (OR). These signals undergo processing to improve the detection of damage-associated characteristics. Initially, the signals undergo rotation by 45 to imitate the placement of the accelerometer in the testing apparatus. Subsequently, both signals undergo normalization to align with the experimental data, which entails subtracting the average and scaling with respect to the standard deviation. Afterwards, the signal envelope is obtained. It was established that the ideal frequency range for envelope calculation varies between the experimental and simulated signals, with 500–1500 Hz being ideal for the simulated data and 1400–2800 Hz for the experimental data.

**Figure 11:**
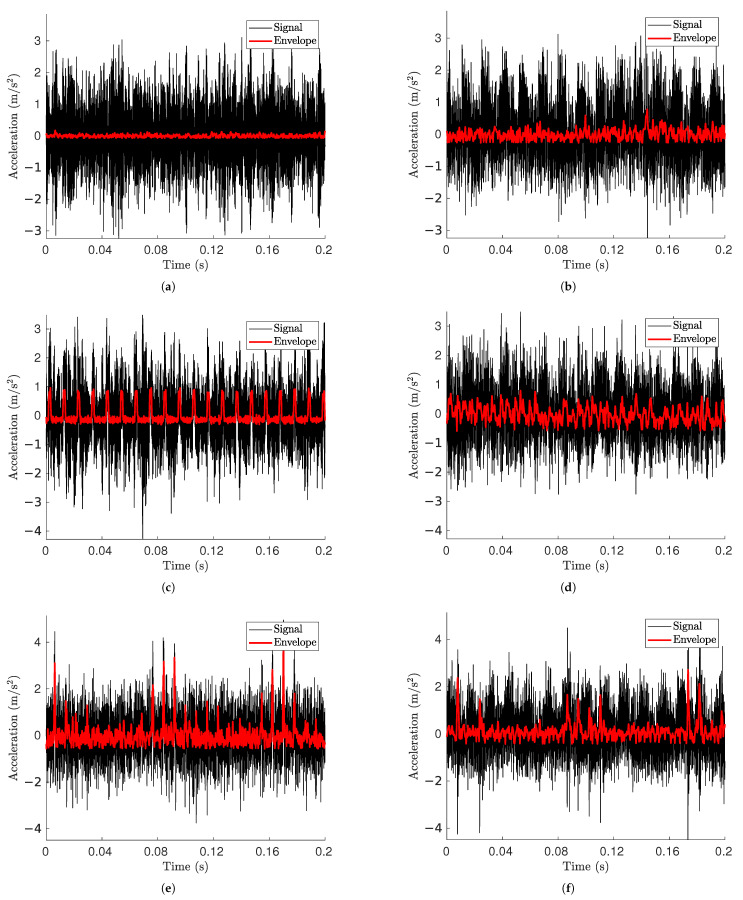
Acceleration signal and its envelope for (**a**) simulated undamaged bearing, (**b**) experimental undamaged bearing, (**c**) simulated OR damage, (**d**) experimental OR damage, (**e**) simulated IR damage, (**f**) experimental IR damage.

The acceleration signals shown in Figure 11 refer to the electric motor’s nominal rotating speed of 727 rpm, which corresponds to a rotating speed of 710 rpm for the shaft, making for a radial load of 124.8 kN acting on the tested bearings. This speed value was used in the simulation for the inner ring rotational speed. Signals from both simulated and experimental signals were normalized with respect to their mean and standard deviation to ensure comparability along the acceleration axis. In addition, it should be noted that it was not the objective of this study to precisely match the amplitude levels, as multiple factors can influence the absolute signal magnitude. These include the specific mounting conditions in the test rig along with the stiffness, damping, and mass of the components. Instead, the model was designed to generate a stable signal where the characteristic fault features are sufficiently distinguishable from other numerical effects [53].

Signals from undamaged bearings (Figure 11a,b) do not reveal apparent signs of fault excitation. The maximum values from the simulation are slightly lower than those from the damaged model, but are still coherent in terms of magnitude. Both of these conditions help to affirm the coherence of the model.

Even with OR damage present (Figure 11c,d), the fault signature is not readily apparent in the acceleration signals from the time domain. Nevertheless, a distinct low-frequency excitation appears after the Hilbert envelope is applied, displaying a periodic pattern that aligns with an OR fault. A significant distinction between the two envelope signals is the increased noise in the experimental data, while the simulated envelope shows a consistent peak pattern that stays constant over time. This is the anticipated behavior for an outer ring fault, where impulses from defects happen with a steady periodicity.

In the case of IR damage, the simulated data (Figure 11e) show the classic pattern consisting of an enveloped signal that spikes every time the inner ring damage contacts an RE, wavering between lower and higher values of the spikes depending on whether or not the fault is in a load zone.

**Figure 12:**
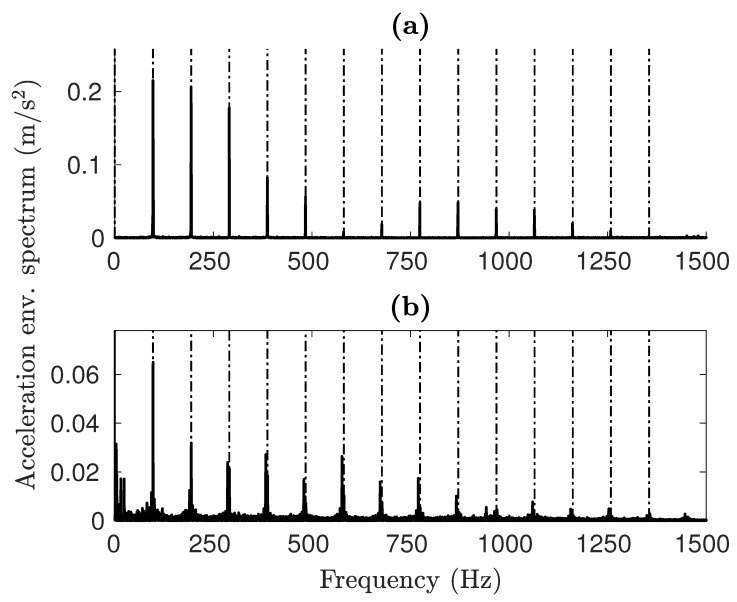
OR fault envelope spectra for (**a**) simulated and (**b**) experimental signals.

The envelope spectrum offers a better understanding of how faults affect the vibrational signals. As illustrated in Figure 12, the results from both simulations and experiments clearly display harmonics of the Ball Pass Frequency Outer (BPFO) type for OR faults. The primary distinction is in the amplitude levels, although the spectral shape stays uniform in both instances. A significant difference is the lack of the fifth harmonic (∼500 Hz) in the simulated spectrum, which appears in the experimental results. This feature is identical to the one obtained from *impulse-train models* [54], where the Fourier transform of a quasi-rectangular function can be assimilated to a Sinc function

(20) $$F\left(\omega \right)\approx \frac{sin(w)}{w}.$$

**Figure 13:**
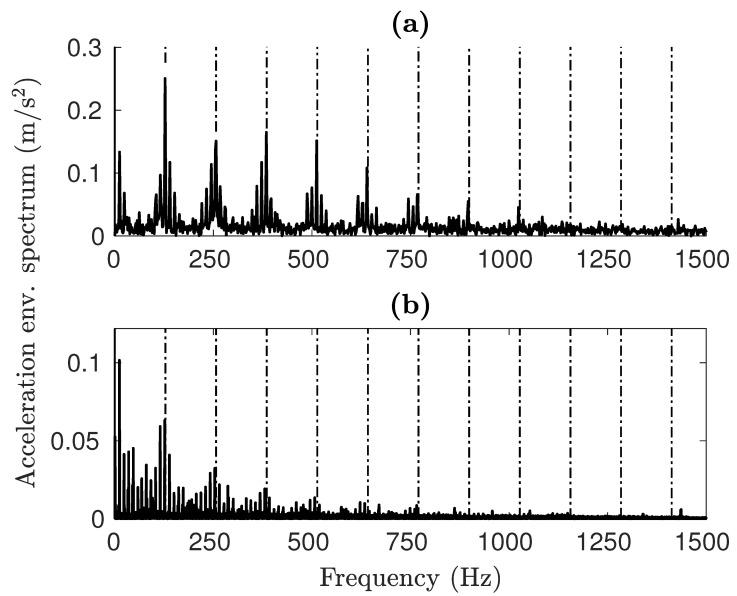
IR fault envelope spectra for (**a**) simulated and (**b**) experimental signals.

Furthermore, low-frequency activity can be seen in the experimental spectra, probably caused by noise. As can be observed in the case of the IR damage shown in Figure 13b, the highest spike is at the carrier frequency framed by the two lower sidebands. The experimental results show the same trend.

Table 6 compares all of the characteristic damage frequencies retrieved from the graphs to the kinematic fault frequencies [2,4]. In both damage cases, the characteristic frequencies identifiable in the spectra of simulated signals are almost identical to the kinematic frequencies. The test rig data with an IR fault deviate the most, with a 1% reduction from the expected value. We attribute this to slippage; as the rollers move more slowly than the analytical speed, the frequency of contact with the fault is reduced as well. The model is very accurate in detecting the excitation frequency from the faults, with a maximum deviation of 0.22% from the analytical data. In general, the model is capable of replicating the damage frequencies with high precision. This validates the model on the objective of correctly representing the dynamic behavior of the bearing.

**Figure 14:**
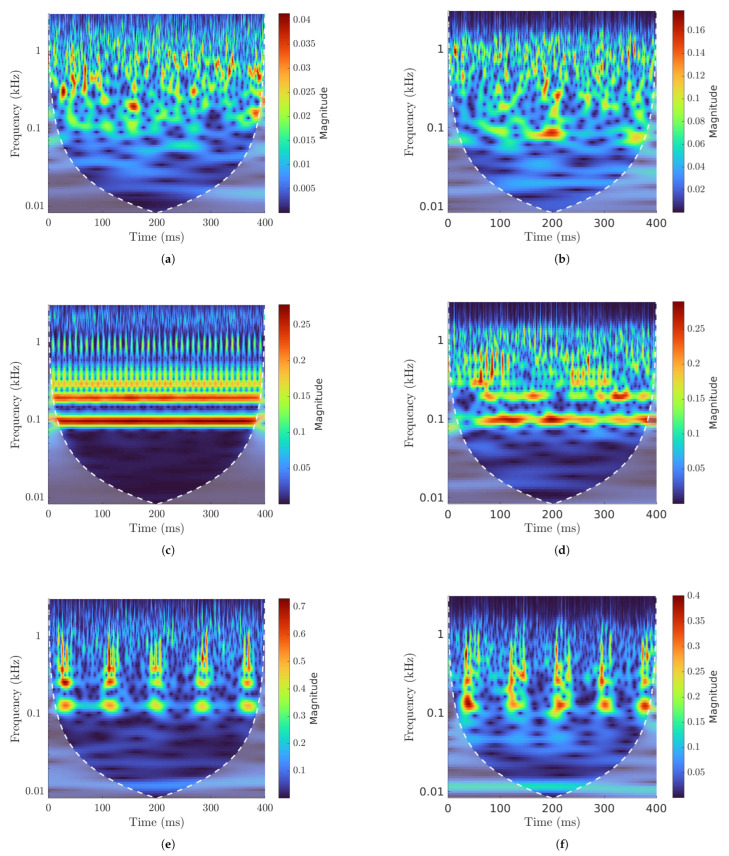
CWT: (**a**) simulated undamaged bearing, (**b**) experimental undamaged bearing, (**c**) simulated OR damage, (**d**) experimental OR damage, (**e**) simulated IR damage, (**f**) experimental IR damage.

Figure 14 shows the CWT for the analyzed signals. Periodicity is not immediately perceivable for undamaged bearings (Figure 14a), where random excitation could prevail. The CWT from an undamaged bearing signal (Figure 14b) is similar to that of the simulated model, and any periodicity cannot be discerned from the scalogram.

Figure 14c shows a clear repetition of impulses with no other type of periodicity. This is the type of graph that characterizes OR faults. Although we do not have enough resolution at the BPFO frequency to see the separation between peaks, we can asses their presence by observing the higher harmonics. CWTs from experimental data show data similar to the simulated results. Indeed, Figure 14d refers to an OR fault, and the periodical spikes in magnitude that occur when the damage meets a RE are identifiable. As in the simulated signal, a periodic excitation cannot be seen clearly at the BPFO frequency, which can instead be observed at higher harmonics. This results are not as clear as the computational ones, as the magnitude of the peaks is not as level but rather changes throughout the run.

Figure 14f shows the IR damage CWT. At this speed, the Ball Pass Frequency Inner (BPFI) for the inner ring fault is 128.04 Hz. Peaks in magnitude for every rotation of the inner ring can be observed, which at the considered speed is every 85 ms. The same resultscan be seen in Figure 14e, except for the BPFI excitation and the added oscillation coming from the rotation of the damage with the IR.

## 5. Discussion

The findings from this research indicate that dynamic simulation of spherical roller bearings can be successfully performed using multibody models generated in the Simscape Multibody environment. Multiple earlier investigations have demonstrated that analysis of smaller bearings can be effectively conducted using models featuring various DOFs. The results presented in this paper indicate that the suggested model is effective for examining more intricate bearing geometries, even when taking into account all DOFs of the components included in the bearing.

A key aspect emphasized by this study is the proposed model’s capacity to precisely replicate the frequency-related phenomena linked to localized damage and its distinct fault frequencies. This factor is especially significant for generating simulated vibration signal databases, which are essential for fault detection and condition monitoring.

Nonetheless, discrepancies in amplitude content have also been observed between the experimental and simulated signals. These variations can be linked to several factors that are not included in the model, such as mounting conditions, damping effects, and the structural flexibility of the test setup. However, comparable constraints have been observed in the literature, suggesting that accurately replicating the signal amplitude continues to be a difficulty in bearing simulations.

The results of this research offer important perspectives for creating vibration-based diagnostic models and strategies for data generation in condition monitoring for heavy industrial applications. Nonetheless, it is important to recognize that attaining high accuracy in the simulation necessitates substantial computational resources, as that the intricacy of the models necessitates advanced hardware infrastructure.

In addition, noise analysis is a relevant aspect of damage diagnostics in industrial settings. However, accurate calculation of signal-to-noise ratios can be challenging when the noise energy is not known a priori. Therefore, the analysis in this study was conducted on numerical signals without introduction of Gaussian-type artificial noise.

A key limitation of the current model is its inability to compute contact pressure distributions due to the discrete point-based representation of one of the contacting surfaces. While methodologies exist for estimating pressure from nodal forces, their implementation would provide only an approximate solution. Future developments could explore the integration of hydrodynamic lubrication models and pressure-based contact force formulations to enhance the accuracy of the contact mechanics representation.

Future efforts should focus on addressing certain existing limitations as well as on broadening the applicability of the model. More specifically, the following elements should be taken into account. The effect of lubrication needs to be incorporated into the model in order to consider its impact on the bearing dynamics and contact forces by means of EHL. This study did not account for defects in the REs, which should be addressed in upcoming simulations to improve the model’s applicability. Because the current model is restricted to radial load situations, broadening it to account for axial loads would enhance its relevance to actual industrial contexts. The results produced by the model could be utilized to train and assess machine learning algorithms for fault identification and predictive upkeep. Additional study is required to determine and separate the sources of inaccuracies in amplitude measurement, as this factor is notably difficult to link with experimental results. Additional efforts will also have to be made regarding the model’s efficiency, as noted before. The authors are aware of both the reasons for the high computational times and of strategies to reduce them. Future developments could also include experiments involving increasing the level of artificial noise in order to evaluate the impact of the presence of noise on the performance of the adopted techniques.

## 6. Conclusions

This work has presented a multibody model for medium to large spherical roller bearings developed in te Simulink^®^ Simscape Multibody environment and validated against experimental data obtained from a dedicated test rig capable of applying significant radial and axial loads. Unlike much of the existing literature, which focuses on smaller bearings, the proposed approach explicitly models all bearing components in a space described with six degrees of freedom. This ensures a more accurate dynamic behavior; in particular, it allows the characteristic fault frequencies for localized inner race and outer race defects to be reproduced with high accuracy.

Our numerical results show close agreement with experimental measurements in the frequency domain, demonstrating the proposed model’s ability to capture defect-induced vibrations. However, amplitude discrepancies remain due to various model assumptions, such as neglected structural compliance, lubrication effects, and certain damping mechanisms. Nonetheless, the model accurately reproduces the fundamental damage signatures, making it a useful tool for generating fault data and aiding in the development or validation of condition monitoring strategies.

Future research could enrich the model by accounting for lubrication phenomena (e.g., elastic–hydrodynamic lubrication), modeling defects on the REs, and broadening load conditions to simultaneously include axial and radial components. Further studies might also investigate the amplitude mismatch issue, whether through refined modeling of the housing structure, more detailed contact formulations, or improved friction/damping parameters. Ultimately, extending these simulations to train and test advanced diagnostic models could further enhance the reliability of vibration-based fault detection methods for heavy industrial applications.

## Figures and Tables

**Table 1:** Fundamental geometry of the bearing.

Variable	Value	Description
$R_{x}$	164 mm	Radius of the raceway in the x direction
α	10°	Base angle of tilt for the REs
$d_{p}$	141.81 mm	Pitch diameter
$T_{RE}$	37 mm	Width of the REs
$d_{RE}$	40.1 mm	Maximum diameter of the REs
$C_{x}$	283.61 mm	Center of the circumference that describes the inner ring raceways
$C_{z}$	50.01 mm	Center of the circumference that describes the inner ring raceways
*M*	19	n. of REs per raceway

**Table 2:** Contact models compatibility matrix.

	CH Solid	Disk	Grid Surface	Infinite Plane	Point Cloud
CH Solid	√	√	x	√	√
Disk	√	×	×	√	×
Grid Surface	×	×	×	×	√
Infinite plane	√	√	×	×	√
Point Cloud	√	×	√	√	×

**Table 3:** HPC hardware characteristics.

CPU	2 × Intel Xeon Scalable Processors Gold 6130 2.10 GHz 16 cores
n. of cores	1824
n. of nodes	57

**Table 4:** Simulation parameters.

Symbol	Parameter	Value
$f_{s}$	Sampling frequency	61,440 Hz
$K_{c}$	Contact stiffness	$5\times 10^{7}$ N/m
$C_{c}$	Contact damping	$10^{3}$ Ns/m
*w*	Limit of smoothing	$10^{-8}$ m
$\mu_{s}$	Static coefficient of friction	0.7
$\mu_{d}$	Dynamic coefficient of friction	0.5
$n_{pc}$	Points per circumference	500
ρ	Density	7850 kg/m^3^
$m_{h}$	Mass of the housing	75 kg
$K_{h}$	Stiffness of the housing	$5\times 10^{9}$ N/m
$C_{h}$	Damping of the housing	$5\times 10^{5}$ Ns/m
$r_{p}$	radial play	0 m
$n_{d}$	Number of damaged points	2

**Table 5:** Load cases and nominal speeds.

	Case 1	Case 2	Case 3	Case 4
Radial load (kN)	0	62.4	124.8	124.8
Axial load (kN)	0	0	0	49
Nominal speeds (rpm)	127, 227, 353, 457, 523, 607, 727, 877, 937, 997			

**Table 6:** Fundamental damage frequencies and percentage variation with respect to kinematic fault frequencies in parenthesis.

Damage Position	Multibody Model (Hz)	Test Rig (Hz)	Kinematic Frequencies (Hz)
OR	96.89 (0.1%)	96.99 (0.2%)	96.79
IR	128.33 (0.22%)	126.76 (−1%)	128.04

## Data Availability

The data presented in this study are available on request from the corresponding author through https://doi.org/10.5281/zenodo.13913254.

## Abbreviations

The following nomenclature is used in this manuscript:

BPFI	Ball Pass Frequency Inner
BPFO	Ball Pass Frequency Outer
CWT	Continuous Wavelet Transform
CH	Convex Hull
cDNAP	Cross-Domain Nuisance Attribute Projection
Degrees of Freedom	DOF
EHL	Elastic–Hydrodinamic Lubrication
GANs	Generative Adversarial Networks
HPC	High-Performance Computing
IR	Inner Race
RF	Reference Frame
RE	Rolling Element
REB	Rolling Element Bearing
STSMM	Security Transfer Support Matrix Machine
OR	Outer Race
WF	World Frame
